# Belly Dancer's Dyskinesia: A Rare Condition

**DOI:** 10.7759/cureus.9604

**Published:** 2020-08-07

**Authors:** Jaimy Villavicencio Kim, Corey Saraceni, Ismail Elkhattib, Lisa Rossi

**Affiliations:** 1 Internal Medicine, University of Connecticut Health, Farmington, USA; 2 Gastroenterology and Hepatology, University of Connecticut, Farmington, USA; 3 Gastroenterology, Saint Francis Hospital, Hartford, USA

**Keywords:** diaphragm, flutter, myoclonus

## Abstract

Belly dancer’s dyskinesia or diaphragmatic flutter (DF) is a rare condition characterized by repetitive involuntary contractions of the diaphragm. Also known as diaphragmatic myoclonus (DM), this disorder can manifest with involuntary movement of the abdominal wall and contraction of accessory respiratory muscles or respiratory myoclonus. Because of its variable presentation, diagnosis can often be difficult and delayed. This phenomenon is thought to be secondary to abnormal excitation of the phrenic nerve, via the central nervous system or along the nerve.Another possible mechanism is the irritation of the diaphragm itself. Diagnosis can be made with ultrasound, thoracic videofluoroscopy, or electromyography (EMG). Different pharmacologic and surgical therapies have been used in the past, but overall, there are no specific guidelines regarding treatment. In this report, we present a case of DF in a young female patient.

## Introduction

Diaphragmatic myoclonus (DM), also known as diaphragmatic flutter (DF), is a rare condition characterized by repetitive involuntary contractions of the diaphragm [1]. Anthonie van Leeuwenhoek first described this entity after suffering from the disorder himself. He described it as a “violent movement” of the diaphragm [1]. DM can manifest with involuntary movement of the abdominal wall and contraction of accessory respiratory muscles, also known as respiratory myoclonus [1,2]. It can also cause a wide range of symptoms including epigastric pulsations, sleep disorders, dyspnea, hyperventilation, hiccups, abdominal pain, acid reflux, and belching [2]. Because of its variable presentation, diagnosis can often be difficult. Additionally, pain provoked by DM can be wrongfully associated with ischemic heart disease [3].

## Case presentation

**Figure 1:**
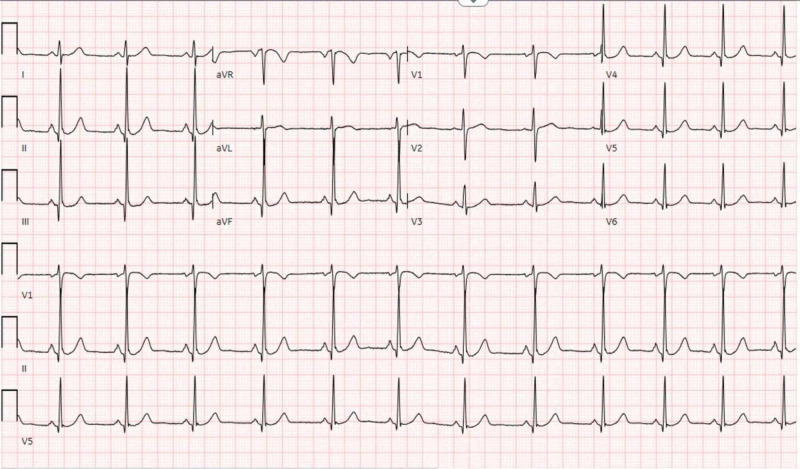
Normal EKG of the patient EKG: electrocardiogram

**Figure 2:**
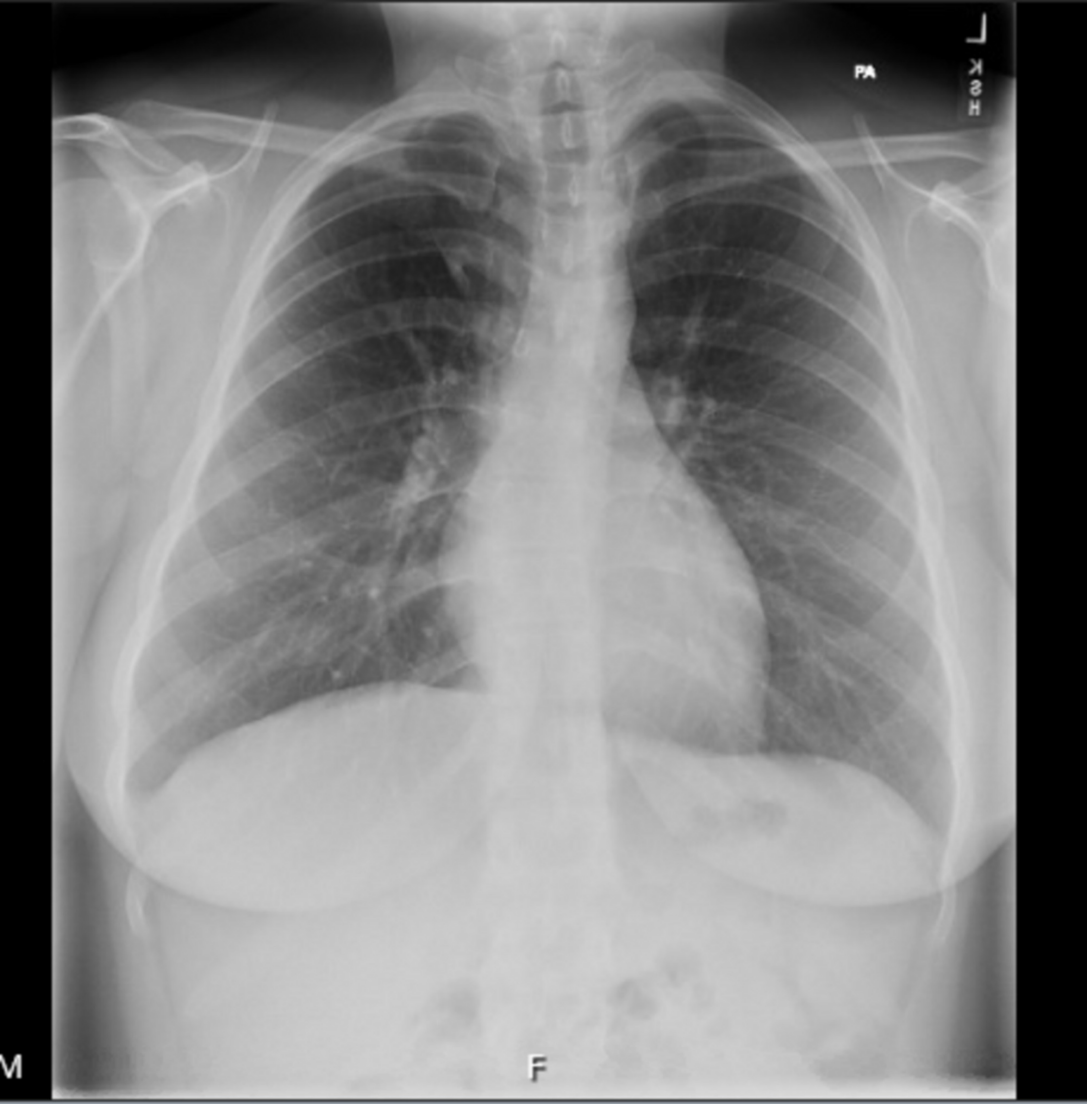
Normal chest X-ray of the patient

**Figure 3:**
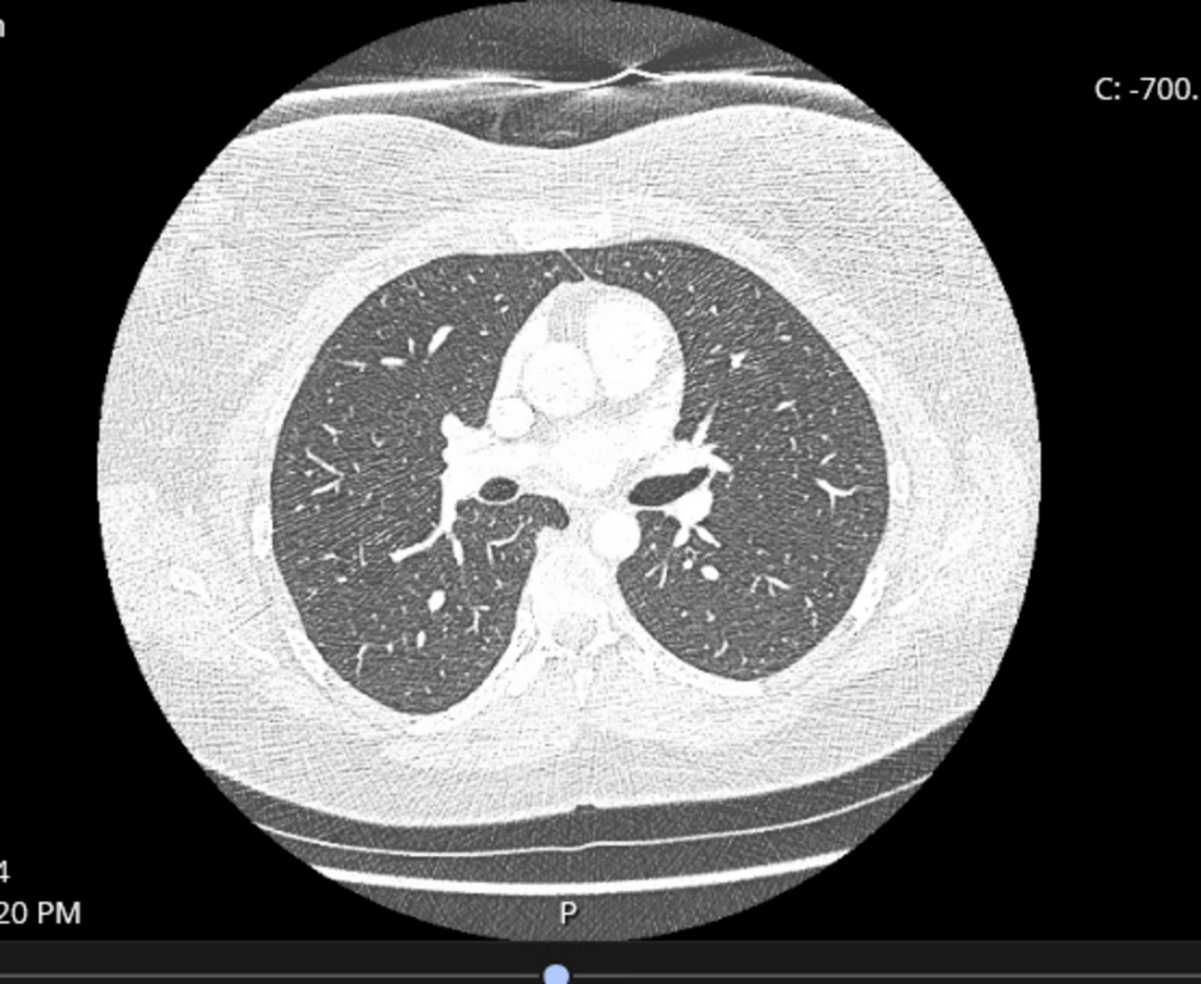
CTA of the chest CTA did not reveal pulmonary embolism CTA: computed tomography angiography

**Figure 4:**
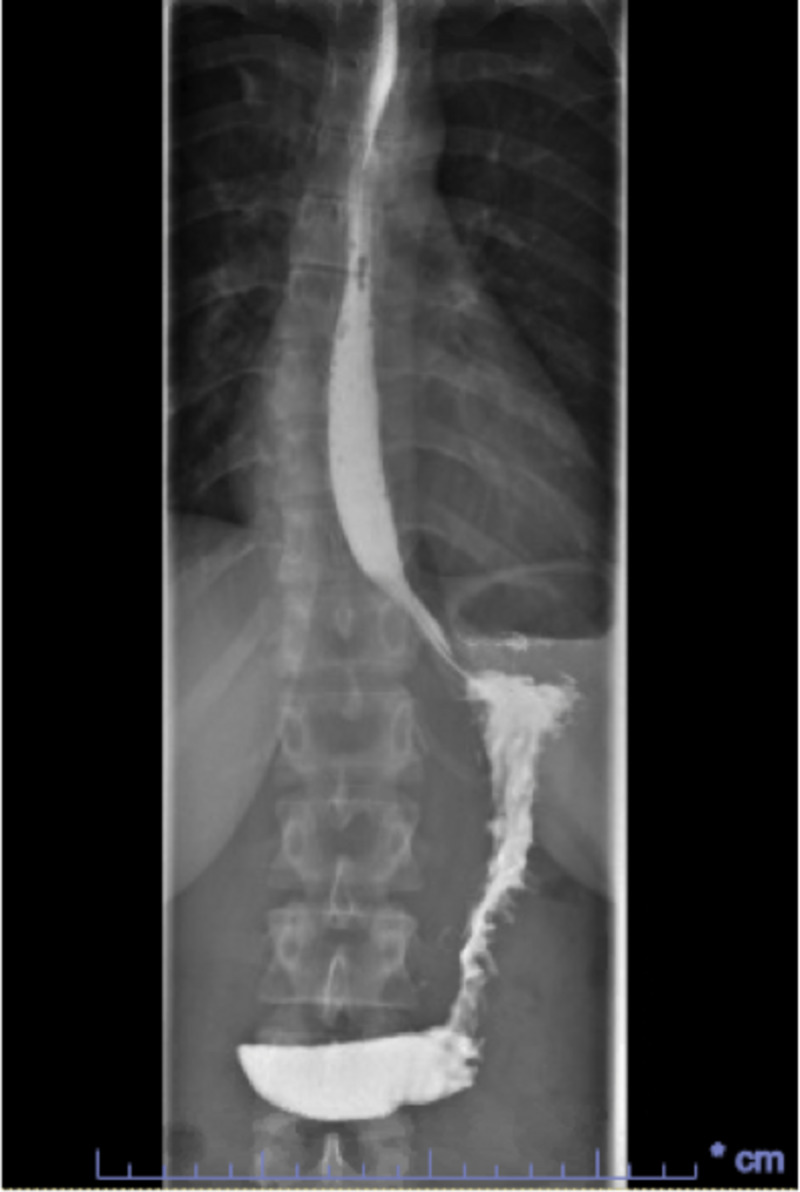
Normal upper GI series of the patient GI: gastrointestinal

A 39-year-old female with a past medical history of depression presented to the hospital due to shortness of breath for the past four days. Her symptom had a sudden start and had worsened with exertion. She had been to an urgent clinic prior to her presentation where she had been prescribed an inhaler, but this had provided no relief. The only other medication she was taking was sertraline, which she had recently increased to a full dose. Electrocardiogram (EKG) (Figure 1) and chest X-ray (Figure 2) were found normal during her initial visit. Bloodwork, including cardiac enzymes, was unremarkable. She was given a nebulizer treatment and discharged home. However, her symptoms persisted, and she went back to the emergency department a day later. Chest X-ray was again normal, and a computed tomography angiography (CTA) ruled out pulmonary embolism (Figure 3). She was started on prednisone, fluticasone, and salmeterol, and once again discharged home. A few days later, an echocardiogram was performed and was found to be normal. Finally, the patient consulted a pulmonologist, who ordered a diaphragmatic ultrasound. This showed a spontaneous involuntary contraction of the right hemidiaphragm with breathing (Video 1). She was ultimately diagnosed with DM or flutter. Of note, she later had an upper gastrointestinal series due to dysphagia, which was normal (Figure 4).

The patient was started on alprazolam without any improvement. She then started to take gabapentin with mild relief of shortness of breath and air hunger. Eventually, she was switched to pregabalin and started on carbamazepine and levetiracetam due to persistent shortness of breath associated with her condition. Her symptoms have subsequently improved, but she is currently undergoing further work-up to rule out organic causes of phrenic nerve irritation.

**Video 1:** Ultrasound of right diaphragm showing involuntary spasms

## Discussion

DM is a rare disorder and is associated with repetitive and involuntary contractions of the diaphragm. The pathophysiology of the condition is not yet well understood and diagnosis remains a challenge. According to Rigatto et al., the pathophysiology behind DM is thought to be abnormal excitation of the phrenic nerve, via the central nervous system or along the nerve [4,5]. Another mechanism described was the irritation of the diaphragm itself [4,5]. Witnessed involuntary movement of abdominal or thoracic muscles can often lead to diagnosis; however, due to the variability of symptoms and presentation, diagnosis remains challenging. In a patient with chest pain, palpitations, or respiratory symptoms with negative cardiopulmonary workup, it might be reasonable to test for DM. This is particularly suggested in young patients and those without any cardiac risk factors.

Diagnosis can be made with thoracic videofluoroscopy or electromyography (EMG) [1]. An EMG can confirm DM by finding a flutter frequency of 0.5-8.0 Hz [1]. Ultrasound offers real-time visualization of the diaphragm and its movements. As seen in our case, this might be a quick, convenient, and non-invasive diagnostic test [2]. Conditions such as encephalitis, stroke, osmotic demyelination, metabolic abnormalities, trauma, and phrenic nerve irritation have been associated with DM [3,4]. It has also been linked to psychogenic disorders and therefore commonly treated with antipsychotics, antidepressants, and/or anticonvulsants. For instance, phenytoin, which stabilizes neuronal membranes and reduces neuronal discharges, has been used with success in the past [3,4,6]. This drug might be useful as it decreases action potentials in the phrenic nerve [3]. Similarly, carbamazepine and other drugs have been effective in other cases [3,6,7]. However, there are no studies to prove the efficacy of any specific agent as data has only been collected from case reports, many of which are decades old. Pharmacologic therapies have been chosen on a case-by-case basis, and have not been consistently effective in all patients.

There are other treatments for DM refractory to pharmacotherapy. Block or transection of the phrenic nerve has been effective in the past [4,6,7]. However, the former method has been associated with recurrence of symptoms, while the latter causes paralysis of hemidiaphragm involved [1]. Phrenic nerve crush has also been reportedly successful in a patient after a failed phrenic nerve block [6]. Botulinum toxin injection with EMG guidance is one of the newer therapies [7]. However, further reports of cases treated with botulinum toxin injection are required to determine the efficacy and appropriate dosing of this medication.

## Conclusions

DM or flutter is an uncommon condition that can present with non-specific symptoms. Due to the rarity of this disorder, there are no guidelines regarding treatment at present. Both pharmacologic and surgical methods have been used in past cases, but choosing one treatment over another might be challenging due to the lack of data concerning effectiveness, side effects, and long-term outcomes.
